# UHRF genes regulate programmed interdigital tissue regression and chondrogenesis in the embryonic limb

**DOI:** 10.1038/s41419-019-1575-4

**Published:** 2019-04-25

**Authors:** Cristina Sanchez-Fernandez, Carlos I. Lorda-Diez, Juan A. García-Porrero, Juan A. Montero, Juan M. Hurlé

**Affiliations:** Departamento de Anatomía y Biología Celular and IDIVAL, Universidad de Cantabria, 39011 Santander, Spain

**Keywords:** Cell death, Cartilage development

## Abstract

The primordium of the limb contains a number of progenitors far superior to those necessary to form the skeletal components of this appendage. During the course of development, precursors that do not follow the skeletogenic program are removed by cell senescence and apoptosis. The formation of the digits provides the most representative example of embryonic remodeling via cell degeneration. In the hand/foot regions of the embryonic vertebrate limb (autopod), the interdigital tissue and the zones of interphalangeal joint formation undergo massive degeneration that accounts for jointed and free digit morphology. Developmental senescence and caspase-dependent apoptosis are considered responsible for these remodeling processes. Our study uncovers a new upstream level of regulation of remodeling by the epigenetic regulators *Uhrf1* and *Uhrf2* genes. These genes are spatially and temporally expressed in the pre-apoptotic regions. UHRF1 and UHRF2 showed a nuclear localization associated with foci of methylated cytosine. Interestingly, nuclear labeling increased in cells progressing through the stages of degeneration prior to TUNEL positivity. Functional analysis in cultured limb skeletal progenitors via the overexpression of either UHRF1 or UHRF2 inhibited chondrogenesis and induced cell senescence and apoptosis accompanied with changes in global and regional DNA methylation. Uhrfs modulated canonical cell differentiation factors, such as Sox9 and Scleraxis, promoted apoptosis via up-regulation of Bak1, and induced cell senescence, by arresting progenitors at the S phase and upregulating the expression of p21. Expression of *Uhrf* genes in vivo was positively modulated by FGF signaling. In the micromass culture assay Uhrf1 was down-regulated as the progenitors lost stemness and differentiated into cartilage. Together, our findings emphasize the importance of tuning the balance between cell differentiation and cell stemness as a central step in the initiation of the so-called “embryonic programmed cell death” and suggest that the structural organization of the chromatin, via epigenetic modifications, may be a precocious and critical factor in these regulatory events.

## Introduction

The autopod is a paddle shaped structure consisting of a core of skeletal progenitors covered by the ectoderm. During the course of development, the progenitors aggregate to form radial digit condensations separated by interdigital regions. Cells in the interdigital regions are initially undifferentiated but soon follow massive degeneration that sculpts the digit contours. In chick and mouse embryos the elimination of the interdigital tissue lasts more than 30 h. During this period the interdigital cells retain stemness to respond to differentiation signals to form ectopic digits^1^.

The control of interdigit remodeling has been the subject of intense research. Morphological and molecular analyses revealed the redundant involvement of several degenerative pathways including canonical apoptosis, cell death associated with lysosomal activation^2,3^, and cell senescence^4^. All these degenerative routes are preceded by intense DNA damage^5^, which is suggestive of common upstream regulation. BMPs have been identified as active factors responsible for interdigit regression. However, while the interdigital application of exogenous BMPs in the embryonic limb induces cell death and DNA damage, the same BMPs stimulate cell proliferation and differentiation in the tip of the fingers, that are also constituted by the same skeletal progenitors^6^. This finding suggests that complementary signals cooperate with BMPs to trigger the degenerative events. The hypothesis of the present study is that the embryonic degenerative processes may share regulatory factors occurring in tumor tissues. DNA damage, cell senescence, and apoptosis are associated with epigenetic and chromatin architecture alterations of cancerous cells^7^ that may determine tumorigenesis and sensitivity to chemotherapy and irradiation^8,9^.

In this study, we analyzed whether ubiquitin-like containing plant homeodomain and RING finger domain (UHRF) epigenetic modulators 1 and 2 participate in the degenerative processes associated with digit development. This gene family is functionally associated with DNA damage and participates in the regulation of proliferation and cell survival of numerous malignancies^10^. UHRF proteins are complex factors containing four functional domains that modulate transcriptional regulation via chromatin modifications. *Uhrf* genes are upregulated in many cancer cells and may behave as either oncogenes or tumor suppressors^10^. Depletion of UHRF1 increases the chemosensitivity of cancer cells to hydroxyurea resistance^11^ and increases their sensitivity to gamma-irradiation^12^. UHRF2, in turn, has been characterized as a component of the ubiquitin proteasome degradation machinery^13^ with pro-apoptotic functions in oncogene-stressed cells^14^. The significance of *Uhrf* genes in developmental systems has received less attention. Mice and zebrafish deficient in UHRF1 die during the course of development^15,16^, and embryonic stem cells null for UHRF1 are hypersensitive to DNA-damaging agents^15^. Furthermore, *Uhrf1* knockout directed to limb mesoderm implicates this protein in appendicular development^17^, as these mice show shortened long bones and dysregulated chondrocyte maturation and proliferation via alterations of the growth plate. *Uhrf2* knockout mice are viable and lack morphological defects^18^, but there is evidence of its implication in the pathogenesis of neurodegenerative diseases^19^. Here, we show that *Uhrf1* and *Uhrf2* genes are expressed in the interdigital mesoderm and interphalangeal joints where undifferentiated cells undergo senescence and apoptosis. At protein level UHRFs associated with zones of DNA methylation. Functional analysis via the overexpression of either UHRF1 or UHRF2 inhibited chondrogenesis and induced cell senescence and apoptosis of cultured limb skeletal progenitors accompanied with changes in global and regional DNA methylation. In a complementary fashion, knockdown of these genes stimulated chondrogenesis and inhibited cell death and senescence. We identified Sox9, Scleraxis, Bak1, and p21 as potential transcriptional targets responsible for its function in the developing digit model.

## Materials and methods

We employed Rhode Island chicken embryos from day 4 to day 8.5 of incubation (id) equivalent to stages 23–34 HH, and C57BL6 mouse embryos ranging from 12 to 14.5 days post coitum (pc).

### In situ hybridization and analysis of cell proliferation

In situ hybridization of PFA-fixed limb specimens was performed in whole mount or 100-μm vibratome sections. The samples were treated with 10 μg/ml of proteinase K for 20–30 min at 20 °C. Hybridization with digoxigenin-labeled antisense RNA probes was performed at 68 °C. Alkaline phosphatase-conjugated antidigoxigenin antibody (dilution 1:2000) was used (Roche). Reactions were developed with BM Purple AP Substrate precipitation (Roche).

The probes for *Uhrf1* and *Uhrf2* were obtained by PCR from RNA extracted from chick or mouse limb buds at initial stages of digit formation. Specific primers for chick *Uhrf1* were: 5′-tccacatctattgcctcaacc-3′ and 5′-gaacaccagattcgctcacc-3′; for chick Uhrf2 5′-agagttcaggtgagcgaagc-3′ and 5′-aggctcaacgtcatctctcc-3 and for mouse Uhrf1: 5′-tgactctggctatggtgtgg-3′ and 5′-gcctgatgttgccgtatagc-3′; and for mouse Uhrf2 5′-agagttcaggtgagcgaagc-3′ and 5′-tcgttcgattccttctgagg-3′.

The distribution of proliferating cells in the autopod was analyzed in paraffin-embedded tissue sections by detection of bromodeoxyuridine (BrdU) incorporation 60 min after injection into the amniotic sac of 100 μl of BrdU solution (100 mg/ml).

### Cell senescence, neutral red vital staining, TUNEL assay, and immunofluorescence

The β-galactosidase activity assay^20^ was performed at pH 6 in vibratome sections of limb autopods fixed in 4% glutaraldehyde.

Neutral red staining, TUNEL assay, and electron microscopy were performed as described previously^2^.

Immunolabeling was performed in limb tissue samples fixed in 4% PFA. We employed both squashed interdigital tissue fragments or vibratome sections permeabilized with Triton X-100 in PBS. The following antibodies were employed: rabbit monoclonal anti-UHRF1 (DSG8E, Cell Signaling), rabbit polyclonal anti-UHRF2 (TA337863, OriGene); mouse monoclonal anti-UHRF2 (sc-398953, Santa Cruz Biotechnology); mouse monoclonal anti-5-methylcytosine (5-mC; 33D3, Eurogentech); rabbit polyclonal anti-SOX9 (AB5535,Milipore); mouse monoclonal anti-γH2AX (JBW301, Milipore-Upstate); and mouse monoclonal anti-BdrU (BU-33, Sigma Aldrich). Counterstaining to distinguish nucleus and cytoplasm was performed using fluorescent-phalloidin (Sigma Aldrich) or DAPI (Vector Laboratories). Observation were made with a LSM51O laser confocal microscope (Zeiss).

### In vivo treatments

The transcriptional effects of FGF signaling in the expression of *Uhrf* genes were studied by analyzing the effects of the local administration of FGF2 (diluted at 0.5 mg/ml, Peprotech), and the FGF inhibitor SU5402 (diluted at 4 mg/ml, Calbiochem), using heparin acrylic (Sigma) or ion exchange (AG1-X2, Bio-Rad) microbeads as described^21^.

### Mesodermal cultures

Dissociated undifferentiated mesoderm from chick leg autopods at 4.5 i.d. (25 HH) were cultured as micromasses containing a density of 2.0 × 10^7^ cells/ml. The chondrogenic outcome was studied under the microscope after Alcian blue staining (0.5% Alcian blue, at pH 1.0). Chondrogenesis was further quantified by the detection of Alcian blue dye extracted in 6 M guanidine–HCl (pH 5.8), and the optical density was measured at 600 nm.

### Cell nucleofection and targeted gene silencing

Functional studies were performed by gain-of-function and loss-of-function approaches.

For gain-of-function experiments, skeletal progenitors were electroporated with constructs of chicken *Uhrf1* (cUhrf1; OGa47434) or *Uhrf2* genes (cUhrf2; OGa21255) cloned into the pcDNA3.1 vector (GenScript) employing the Multiporator System (Eppendorf) and cultured under high-density conditions as indicated above. For loss-of-function experiments, skeletal progenitors were electroporated with a short hairpin RNAi against *Uhrf1* (*sh-Uhrf1*) or *Uhrf2* (*sh-Uhrf2*) cloned into the pcU6–1-shRNA (a generous gift from Dr. Tim J. Doran). Transfections with the respective empty plasmids were employed as controls. After 48 h of culture, the level of gene regulation was confirmed by q-PCT and/or Western blot analysis.

### Evaluation of DNA methylation

Changes in global methylation after Uhrf functional experiments were determined by the ELISA-based commercial kit Imprint^®^ Methylated DNA Quantification (MDQ1; Sigma-Aldrich, St. Louis, MO, USA). One hundred and fifty nanograms of genomic DNA from our samples were incubated with capture and detection antibodies and their absorbance was measured at 450 nm. The amount of methylated DNA present in the samples is proportional to the absorbance measured. Quantification of global DNA methylation was performed calculating methylation levels relative to the methylated control DNA (50 ng/µl) using the formula: [(A450 av sample–A450 av blank)/(A450 av methylated control DNA–A450 av blank)] × 100.

We next selected Bak1 as potential target of Uhrf genes in the control of cell death. Changes in the methylation status of its promoter were studied by methylation sensitive restriction enzyme and quantitative polymerase chain reaction (MSRE-qPCR). Primers were designed using Primer3Plus online software. The selected PCR primer pair flanked the region of interest (based on the presence of informative restriction sites) within the promoter of Bak1: Fwd, AGCTGCAGCCTTCCCAGA; Rev, CTCTAGAGGCGCCTTGCAC. Genomic DNA samples were digested with a CpG-methylation-sensitive restriction enzyme (TauI (3 U/µl); ER1651 ThermoFisher Scientific) or with a non-CpG-methylation-sensitive enzyme (SacI (10 U/µl); ER1132 ThermoFischer Scientific) for 2 h according to the manufacturer's suggested temperature. SYBRGreen-based qPCR was carried out in triplicates with a total volume of 20 µl per tube containing 1 µl of genomic DNA (TauI-digested, SacI-digested, or undigested DNA), 0.4 µl of each primer, 10 µl of SYBR Select Master Mix (Life Technologies), and 8.2 µl of H_2_O. Reactions were carried out in a StepOne Real Time System and analyzed by StepOne software v2.3 (Life Technologies). The relative percentage of methylated DNA was calculated according to the equation 2^−∆ΔCt^ employing as normalizers the Ct values of both SacI digested and undigested samples^22^.

### Western blot analysis

The total amount of UHRF proteins in control and treated tissue samples was evaluated by Western blot analysis. Total proteins were extracted by lysis from interdigits or dissociated micromass cultures. After determining the protein concentration, 30 µg of each sample was loaded onto a 12.5% SDS polyacrylamide gel, electrophoresed and transferred to PVDF membranes. The membranes were incubated with primary antibodies (see “Immunofluorescence” section). Protein bands were detected with an Odyssey^TM^ Infrared-Imaging System (Li-Cor Biosciences). Immunoblots were developed with anti-mouse IRDye800DX or anti-rabbit IRDye680DX as secondary antibodies (Rockland Immunochemicals, USA).

### Flow cytometry

Control, *Uhrf1* or *Uhrf2* gene-overexpressing, or *Uhrf1* or *Uhrf2* gene-silenced cultures were dissociated. One million cells were used in each test. For propidium iodide (PI) staining, the cells were washed with PBS and fixed in 90% ethanol. The samples were incubated overnight at 4 °C with 0.1% sodium citrate, 0.01% Triton X-100, and 0.1 mg/ml PI. The cell suspension was subjected to flow cytometry analysis in a Cytoflex (Beckman Coulter) and analyzed with the Cytexpert software.

### Real-time quantitative PCR (q-PCR) for gene expression analysis

Total RNA was extracted using the NucleoSpin RNA kit (Macherey-Nagel). First-strand cDNA was synthesized using random hexamers and the High Capacity cDNA Reverse Transcription Kit (Life Technologies). The cDNA concentration was adjusted to 0.5 μg/μl. SYBRGreen (Life Technologies)-based q-PCR was performed using the Mx3005P system (Stratagene). Rpl13 was chosen as the normalizer in interdigital samples and Gapdh in cultures. Mean values for fold changes were calculated. Expression level was evaluated relative to a calibrator according to the 2^−(ΔΔCt)^ equation. Each value represents the mean ± SEM of at least four independent samples obtained under the same conditions. Data were analyzed using Student’s *t*-test or ANOVA followed by Bonferroni test for post-hoc comparisons. Statistical significance was set at *p* < 0.05. q-PCR-specific primers analyzed in this study would be provided upon request.

## Results

### Interdigital and interphalangeal joint expression domains of UHRF1 and UHRF2 in the embryonic limb

**Fig. 1:**
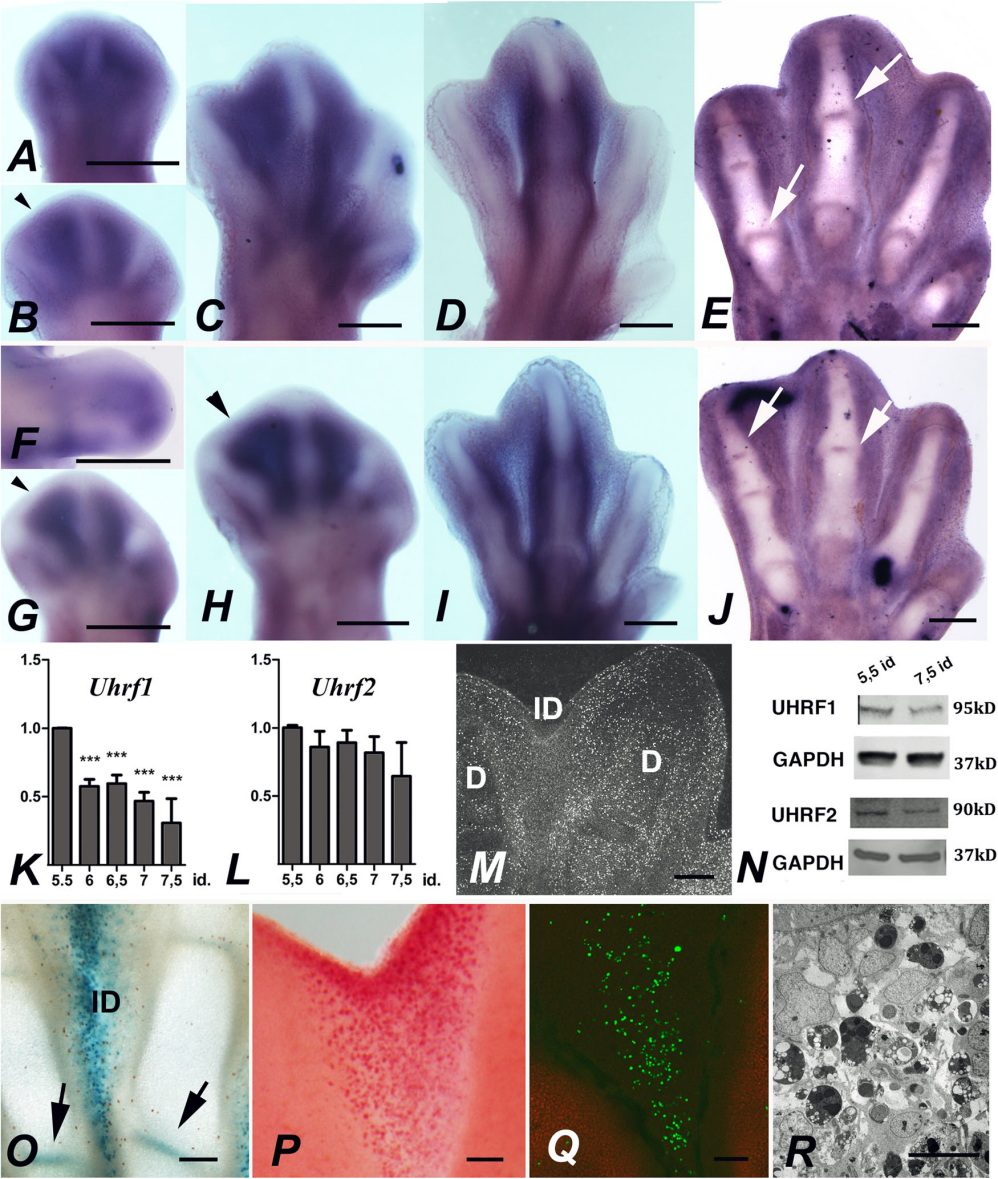
Uhrf genes are expressed in the remodeling interdigits. Expression of *Uhrf1* (**a**–**e**) and *Uhrf2* (**f**–**j**) in the interdigital tissue and joint forming regions of chick leg buds.Whole mount in situ hybridizations at id 5 (**a**), 4.5 (**f**), 5.5 (**b** and **g**), 6.5 (**c**, **h**), and 7.5 (**d**, **i**). Arrowheads in **g** and **h** show a characteristic absence of Uhrf2 transcripts in the subridge mesoderm. **e** and **j** are in situ hybridizations in vibratome tissue sections at id 7.5 to show the joint domains (arrows) of *Uhrf1* (**e**), and *Uhrf2* (**j**). **k** and **l** are charts showing the expression levels of *Uhrf1* (**k**) and *Uhrf2* (**l**) in the course of interdigit tissue remodeling, considering a value of 1 for id 5.5. Note the decreased expression of *Uhrf1* between id 5.5 and 6. **m** BrdU incorporation to mark mitosis in the autopod at id 6. Note the almost absence of cells labeled in the interdigital tissue (ID) compared with the digit regions (**d**). **n** Western blotting showing the UHRF1 and UHRF2 proteins in the interdigital tissue at id 5.5 and 7.5 the stage preceding and the peak of degeneration. **o**–**r** illustrations of the most characteristic features of the third interdigit in autopods at the peak of degeneration (id 7.5). **o** Senescence-specific beta-galactosidase activity. Note positivity in the interdigit (ID) and in the developing joints (arrows); **p** interdigit after neutral red vital staining; **q** immunolabeling with anti-γ-H2AX showing interdigital cells undergoing DNA damage. Digit rays are labeled red with anti-Sox9; **r** transmission electron microscopic image to show the abundance of dark apoptotic cells in the interdigital mesoderm. ***p < 0.001. Bars = 200 µm (**a**–**j**, **m**, **o**–**q**). Bar = 15 µm (**r**)

A preliminary search for the interdigital expression of epigenetic regulators revealed high expression levels of *Uhrf1* and *Uhrf2* genes compared with interdigital gene markers^23^ (Supplementary Fig. 1). Therefore, we generated probes to explore their expression by in situ hybridization. As shown in Fig. 1a–j, *Uhrf* genes show similar expression pattern in the interdigital mesoderm, preceding (id 5–6) and during the whole remodeling period (id 6.5–8; Fig. 1a–j). This period is characterized by proliferation arrest^24,25^(Fig. 1m), and degeneration detectable by β-galactosidase labeling (Fig. 1o), neutral red vital staining (Fig. 1p), γH2AX immunolabeling (DNA damage marker; Fig. 1q), and TEM (Fig. 1r). Analysis of the gene expression in tissue sections (Fig. 1e, j) detected additional domains in the developing interphalangeal joints that mark a region where cells also undergo programmed cell death and senescence (Fig. 1o). Quantification of interdigital gene expression showed that *Uhrf1*, after a short downregulation preceding id 6, maintained in subsequent stages expression levels higher than other interdigital tissue markers (Fig. 1k, see also Supplementary Fig. 1). In contrast, the expression level of *Uhrf2* remained relatively constant throughout the course of tissue remodeling (Fig. 1l). Western blot analysis confirmed the presence of high levels of UHRF1 and UHRF2 proteins through the course of interdigit remodeling (Fig. 1n).

**Fig. 2:**
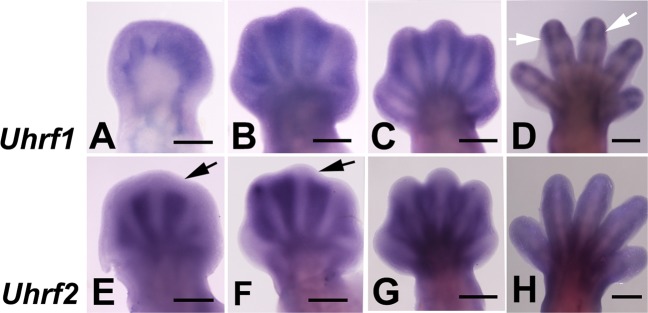
In situ hybridizations showing the expression of *Uhrf1* (**a**–**d**) and *Uhrf2* (**e**–**h**) in the mouse autopod during interdigit remodeling. **a**–**d** show the expression of Uhrf1 at pc days 12 (**a**), 13 (**b**), 13.5 (**c**), and 14 (**d**). Note the presence of joint domains by pc 14 (arrows in **d**). **e**–**h** show the expression of *Uhrf2* at pc days 12.5 (**e**),13 (**f**), 13.5 (**g**), and 14 (**h**). Note the fading of the interdigital domains in the distal subectodermal region (arrows in **e** and **f**). Bars = 500 µm

A similar expression pattern for both genes was observed in mouse embryos. As shown in Fig. 2, by day 12 pc, *Uhrf* genes are expressed in the undifferentiated autopodial mesoderm and in subsequent stages, expression becomes restricted to the interdigital mesoderm. By day 14 pc, interdigit regression is almost accomplished and *Uhrf* gene expression become restricted to the zones of joint formation (Fig. 2d, h)

### Cellular distribution of UHRF in the interdigital mesoderm

**Fig. 3:**
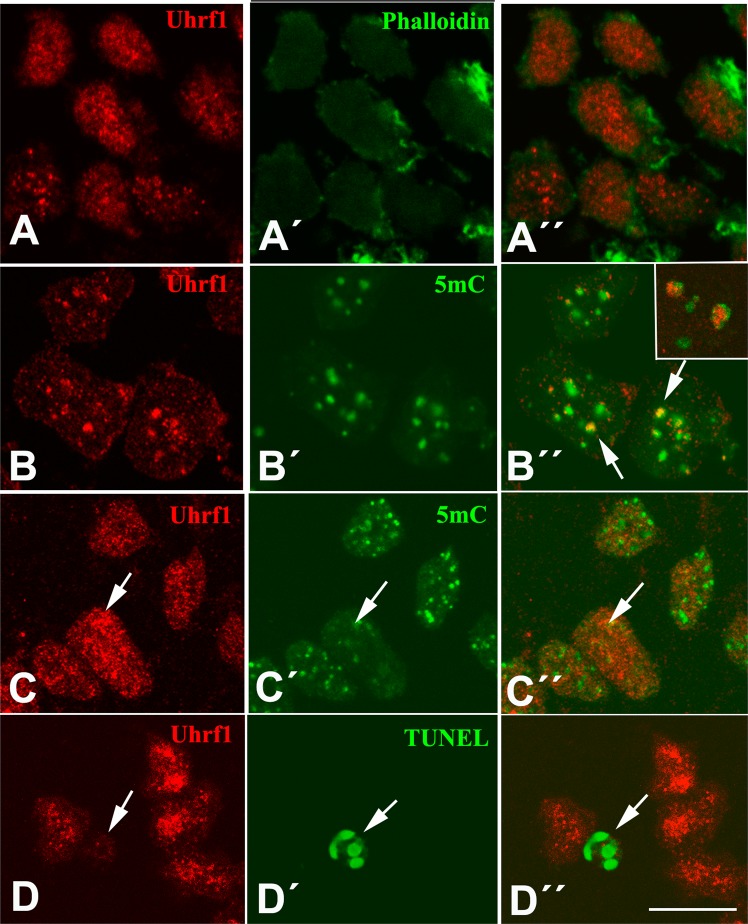
UHRF1 immunolabeling (red) of chick interdigital cells at 7 id. **a**–**a**´´ UHRF1 (red) in combination with phalloidin (green) show the nuclear localization of UHRF1. (**a**) UHRF1 labeling, (**a**´) phallodin labeling showing cytoplasmic actin; (**a**´´) merged images. (**b**–**b**´´) UHRF1 (red) in combination 5-mC immunolabeling (green) to show the overlapping distribution of UHRF1 in zones of DNA methylation (arrow). (**b**) UHRF1 labeling; (**b**´) 5-mC immunolabeling; (**b**´´) merged images; inset in this image shows the association between UHRF1 and 5-mC rings in mouse interdigital cells. (**c**–**c**´´) Double immunolabeling for UHRF1 (red) and 5-mC (green) to show the increased UHRF1 labeling in cells with reduced DNA methylation (arrows). (**c**) UHRF1 labeling; (**c**´) 5-mC immunolabeling; (**c**´´) merged image. (**d**–**d**´´) Immunolabeling of UHRF1 (red) in combination with TUNEL (green) to show the loss of UHRF1 in TUNEL-positive apoptotic cells (arrows). (**d**) UHRF1 labeling; (**d**´) TUNEL labeling; (**d**´´) merged image. Bar = 10 µm

**Fig. 4:**
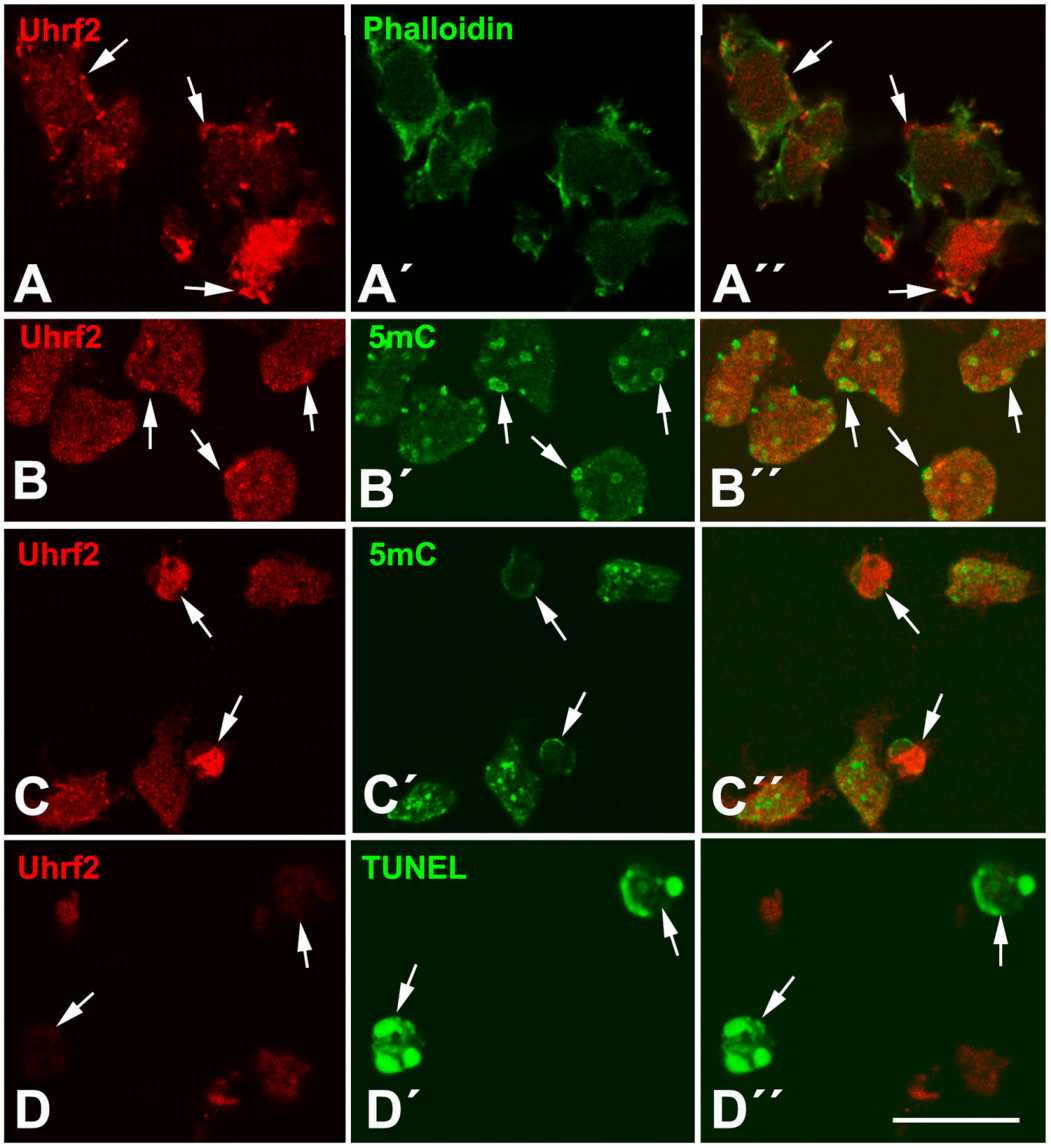
UHRF2 immunolabeling of interdigital cells. UHRF2 immunolabeling (red) of chick (**a**, **c**, **d**) and mouse (**b**) interdigital cells.(**a**–**a**´´) UHRF2 (red) in combination with phalloidin (green) showing the occurrence of both nuclear and cytoplasmic (arrow) localization of UHRF2. (**a**) UHRF2 labeling, (**a**´) phallodin labeling showing cytoplasmic actin; (**a**´´) merged images. (**b**–**b**´´) UHRF2(red) in combination 5-mC immunolabeling (green) to show the presence of UHRF2 positivity in the core of 5-mC rings (arrows). (**b**) UHRF2 labeling; (**b**´) 5-mC immunolabeling; (**b**´´) merged images. (**c**–**c**´´) Double immunolabeling for UHRF1 (red) and 5-mC (green), to show the increased immunolabeling in cells with reduced DNA methylation (arrows). (**c**) UHRF2 labeling; (**c**´) 5-mC immunolabeling; (**c**´´) merged image. (**d**–**d**´´) Immunolabeling of UHRF1 (red) in combination with TUNEL (green) to show the loss of UHRF2 in apoptotic cells TUNEL-positive (arrows). (**d**) UHRF1 labeling; (**d**´) TUNEL labeling; (**d**´´) merged image. Bar = 10 µm

The protein distribution was analyzed by immunofluorescence in mouse and chick interdigits (Figs. 3 and 4). Both proteins showed a diffuse pattern throughout the nucleus with distinctive foci of labeling intensification (Figs. 3a and 4a). Moderate cytoplasmic immunolabeling was also observed (Fig. 4a). The association of UHRF proteins with epigenetic markers and degenerative events was next explored (Figs. 3b–d and 4b–d). We first analyzed the relation between UHRF proteins and methylated DNA monitored with 5-mC immunolabeling. In chick cells, 5-mC immunolabeling appeared as dots of variable size (Fig. 3b´ and c´, and 4c´). In mouse cells, 5-mC labeling appeared as characteristic rings (Fig. 4b´). In both species UHRF1 and UHRF2 spots were often associated with 5-mC-positive marks. Of note, UHRF proteins often occupied the core of the 5-mC rings in mouse cells (inset of Fig. 3b´´, and Fig. 4b´´). Colocalization of UHRF proteins with 5-mC foci were appreciated for UHRF1 (Fig. 3b). This overlapping expression is consistent with the demonstrated role of UHRF1 in recruiting DNA methyl transferase 1 and histone deacetylase 1 to chromatin regions containing CpG dinucleotides. Of relevance, immunolabeling for both UHRF1 and 2 appeared intensified at the initial stages of the dying process (Figs. 3c and 4c). These initial stages of cell degeneration are identified by a progressive loss and peripheral displacement of 5-mC labeling. In contrast, at stages of overt apoptosis UHRF positivity was lost (Figs. 3d and 4d).

### Regulation of *Uhrf* gene expression by FGF signaling

**Fig. 5:**
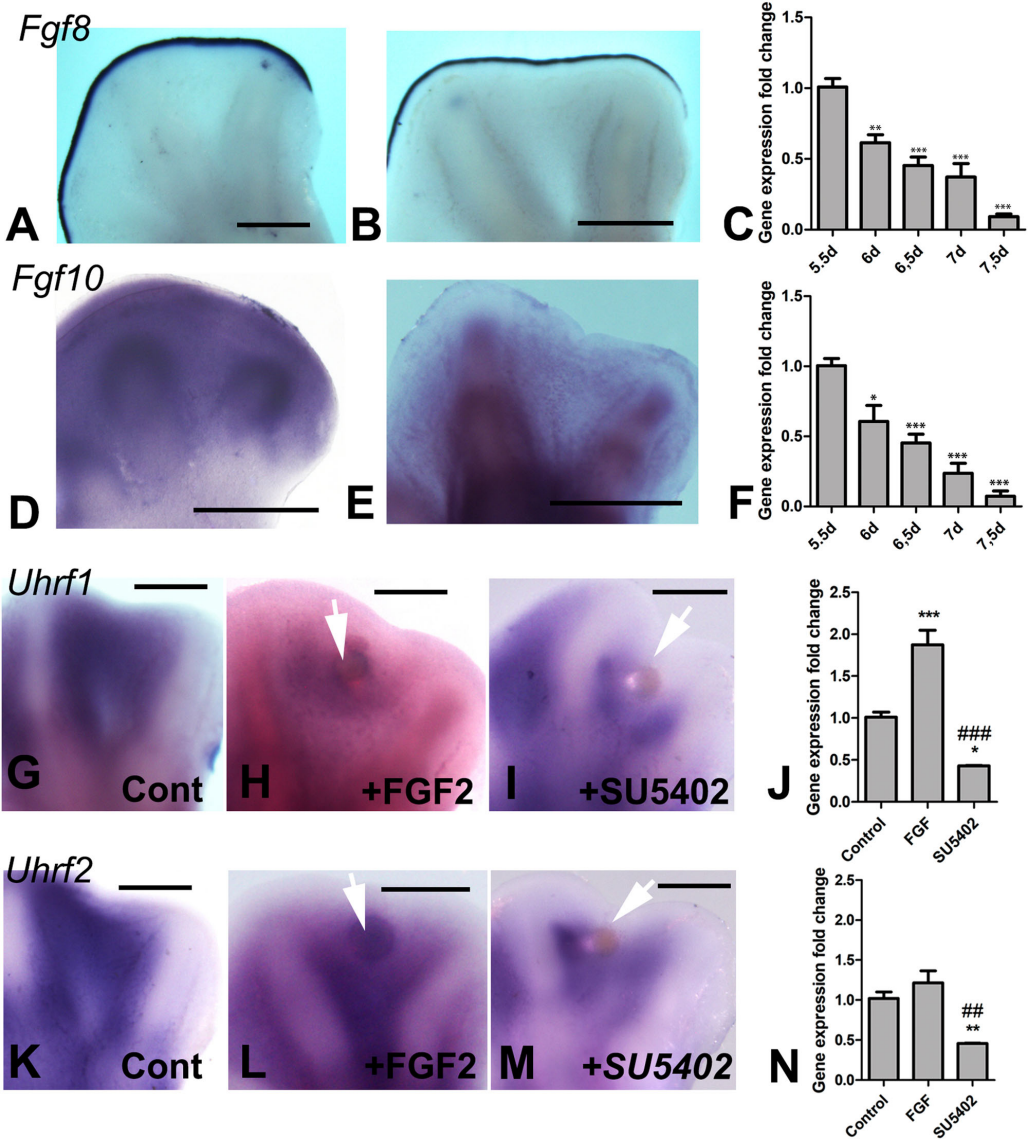
Regulation of Uhrf genes by FGF signaling. **a, b** Expression of *Fgf8* in the limb marginal ectoderm (AER) at id 5.5 (**a**) and 7 (**b**). **c** q-PCR analysis of the expression decay of *Fgf8* gene expression from 5.5 to 7.5 id in samples of the interdigital tissue. **d**, **e** expression of *Fgf10* in the autopods at id 5.5 (**e**) and 7,5 (**f**). **f** q-PCR analysis of the expression decay of *Fgf10* gene expression from 5.5 to 7.5 id in samples of the interdigital tissue. **g**–**j** Regulation of *Uhrf1* by FGF signaling. **g**–**i** expression of *Uhrf1* in control interdigit at id 7.5 (**g**); 12 h after implantation of a FGF-bead (arrow, **h**); and 12 h after implantation of a SU5402 bead (arrow, **h**). **I** q-PCR analysis of the regulation of *Uhrf1* in control interdigits and 12 h after implantation of a FGF2 bead and SU5402 beads. **k**–**n** Regulation of *Uhrf2* by FGF signaling. **k**–**m** expression of *Uhrf2* in control interdigit at id 7.5 (**k**); 12 h after implantation of a FGF-bead (arrow, **l**); and 12 h after implantation of a SU5402 bead (arrow, **m**). **n** q-PCR analysis of the regulation of *Uhrf2* in control interdigits and 12 h after implantation of a FGF2 bead and SU5402 beads. ***p < 0.001; **p < 0.01; *p < 0.05 versus control. ^###^*p* < 0.001; ^##^*p* < 0.01 between the two treatments. Bars = 200 µm

The growth and differentiation of the limb skeletal progenitors is controlled by FGFs. FGFs maintain proliferation and delay cell death and chondrogenic differentiation^21,26–28^. The degeneration of the interdigital tissue and the completion of digit formation are associated with the extinction of FGF signaling. Therefore, we investigate the influence of FGFs on the expression of UHRF genes. As shown in Fig. 5a–f, the expression levels of FGF8 and FGF10, which are the most characteristic *FGF* genes expressed in the autopod at the studied stages, become downregulated at id 6 (Fig. 5a–f). As described above (Fig. 1k), id 6 is the period that marks the decreased expression of *Uhrf1*. In subsequent stages, when downregulation of FGFs is intensified, both *Uhrf1* and *Uhrf2* remain expressed at high levels. Interdigital application of a FGF-bead moderately upregulated the expression of *Uhrf1* (Fig. 5g, h and j), but not *Uhrf2* (Fig. 5k–l and n). In contrast, the local application of the FGF inhibitor SU5402 downregulated both *Uhrf* genes (Fig. 5i, j, and m, n). Together, these findings suggest that FGF signaling promotes only the expression of *Uhrf1* but sustains the expression of both *Uhrf* genes.

### Expression and functional analysis of *Uhrf* genes during chondrogenic differentiation

**Fig. 6:**
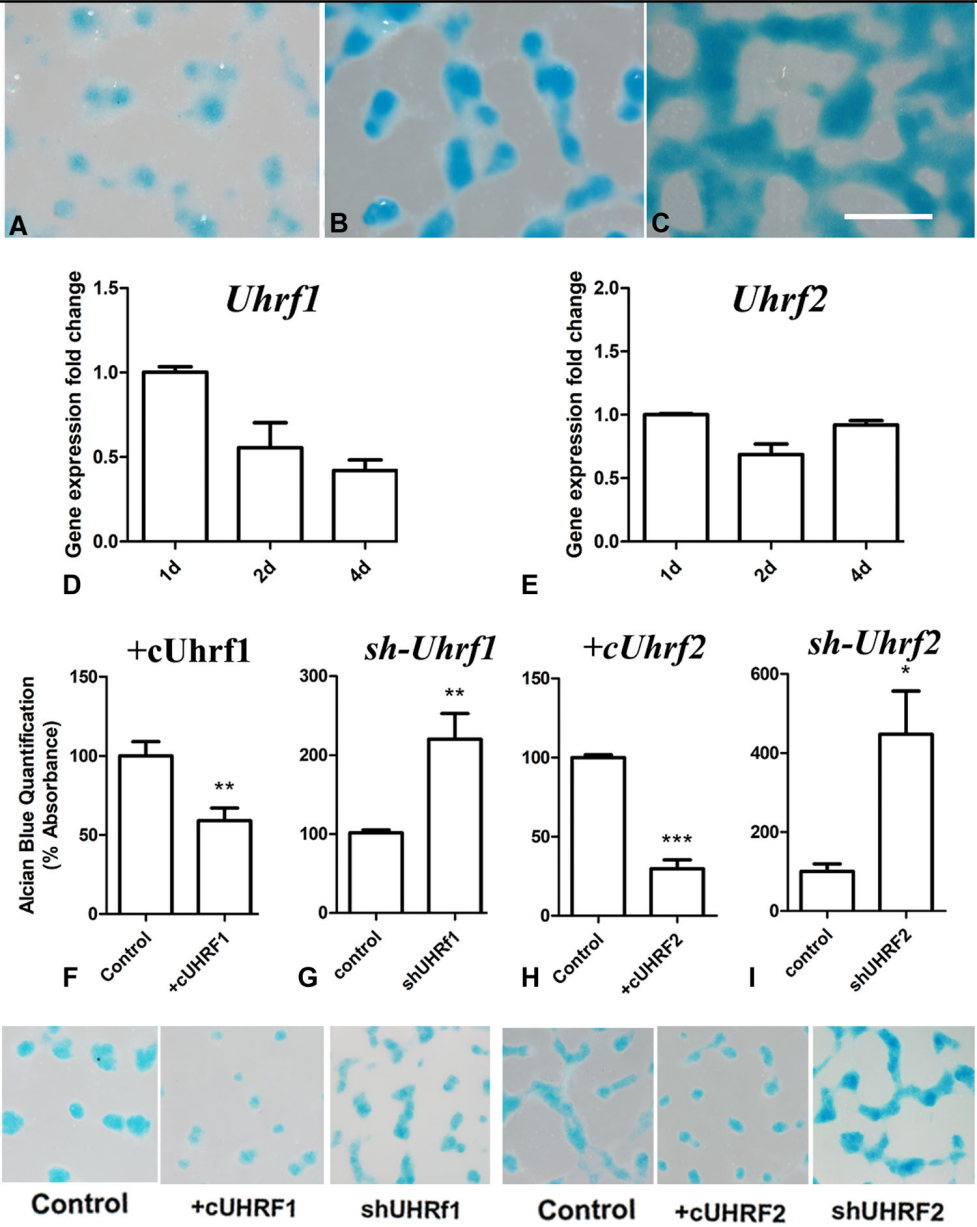
Uhrf genes and chondrogenesis of limb skeletal progenitors. **a–c** Limb skeletal progenitors cultured at high density (micromass) for 2 (**a**), 3, (**b**) and 4 (**c**) days stained with Alcian blue for chondrogenesis. Note the increase in the size of the cartilage nodules stained in blue. Bar = 200 µm. **d** q-PCR quantification of *Uhrf1* gene expression in micromass cultures of 1, 2, and 4 days. Note the downregulation of *Uhrf1* during the course of chondrogenic differentiation. **e** q-PCR quantification of *Uhrf2* gene expression in micromass cultures of 1, 2, and 4. In contrast to *Uhrf1*, *Uhrf2* is not downregulated in the differentiating cultures. **f–i** evaluation of cartilage differentiation by guanidine–HCl extraction of Alcian blue dye (illustrated in the lower row of pictures) in functional experiments of *Uhrf* genes gain-of-function (**f** and **h**) and loss-of-function (**g** and **i**). Note the inhibition of differentiation after the overexpression of *Uhrf1* (**f**) and *Uhrf2* (**h**), and the increased chondrogenesis after knockdown of *Uhrf1* (**g**) and *Uhrf2* (**i**). ***p < 0.001; **p < 0.01; *p < 0.05 treated versus control

The absence of *Uhrf* transcripts in the differentiating digit rays revealed these factors as potential inhibitors of chondrogenic differentiation. To test this hypothesis we monitored their expression in micromass cultures of limb skeletal progenitors (Fig. 6). This in vitro assay mimics the process of digit cartilage differentiation in vivo. The skeletal progenitors grow initially in an undifferentiated state, but by 48 h of culture, they aggregate to form chondrogenic nodules (Fig. 6a). The nodules are separated from each other by cells that retain morphological and transcriptional characteristics of fibrous connective tissue^29^. In subsequent days of culture, the chondrogenic nodules increased in number and size (Fig. 6a–c), forming by the end of the second week an almost continuous sheet of cartilage^30^. *Uhrf1* expression showed the highest levels on day 1 of culture, but the expression declined to half by day 2, progressing to more moderate levels during subsequent days of culture (Fig. 6d). At difference of *Uhrf1*, *Uhrf2* maintained uniform expression levels during the first 4 days of culture (Fig. 6e). These results are compatible with an antichondrogenic role, at least for *Uhrf1*.

To further explore their potential role in chondrogenesis, a functional analysis of *Uhrf* genes was performed by gain-of-function and loss-of-function experiments in the micromass assay (Fig. 6f–i). Chondrogenesis was significantly downregulated in 5-day cultures of progenitors transfected with either *Uhrf1* (Fig. 6f) or *Uhrf2* (Fig. 6h) genes. In a complementary fashion, chondrogenesis was significantly upregulated after the knockdown of either *Uhrf1* (Fig. 6g) or *Uhrf2* (Fig. 6i), via transfection of the corresponding sh-RNAi.

### *Uhrf* genes modulate cell death and cell cycle progression of skeletal progenitors

**Fig. 7:**
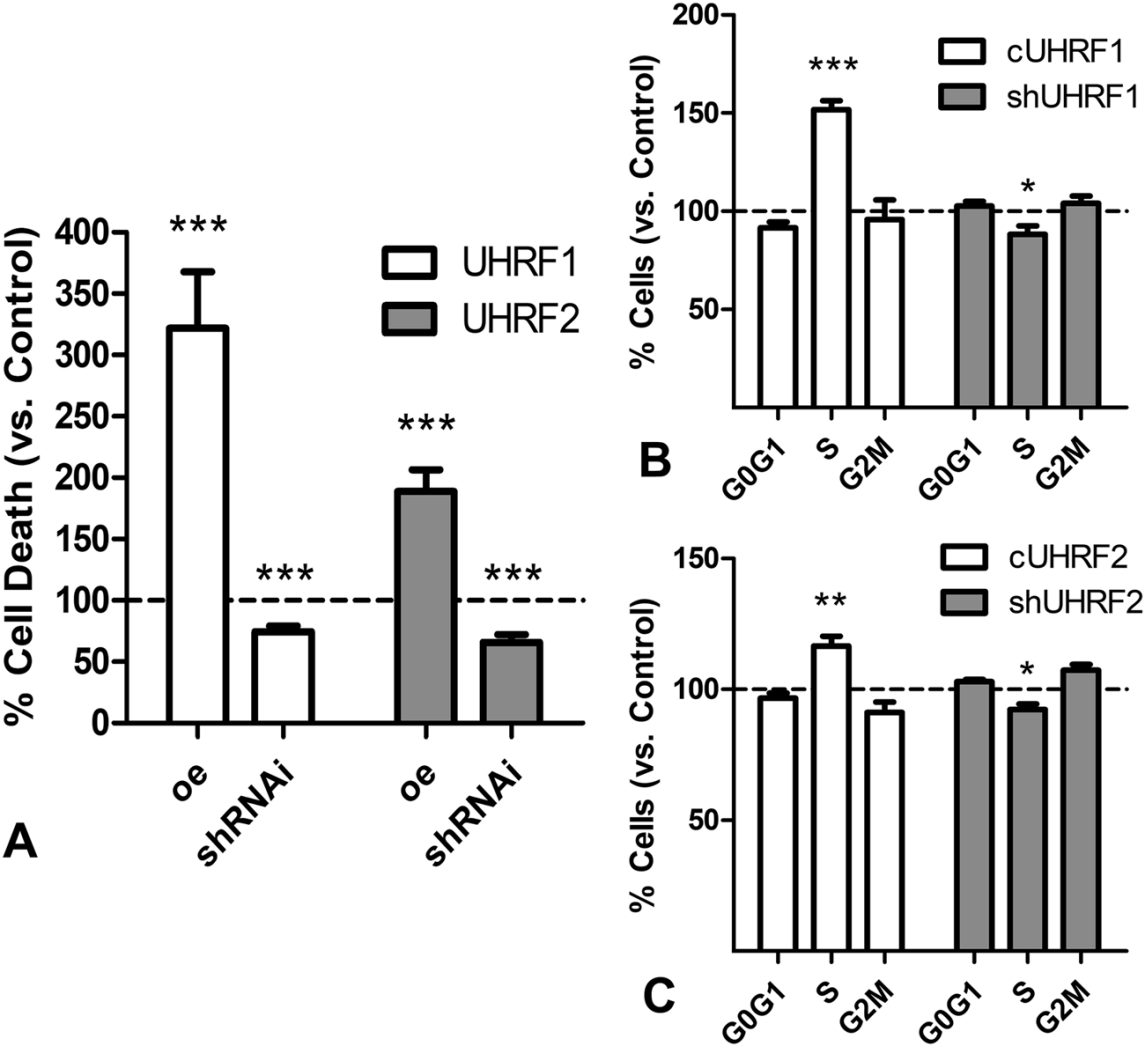
Regulation of cell death and cell cycle by UHRFs. **a** Chart showing differences in the intensity of cell death evaluated by flow cytometry between control progenitors and progenitors overexpressing (oe) or subjected to silencing (shRNAi) of *Uhrf1* (white columns) and *Uhrf2* (gray columns).The level of cell death in control cultures was considered 100% and is represented by the dotted line. **b** Graphic illustrations comparing the proportion of cells at different cell cycle stages in cultures overexpressing *Uhrf1* (white columns) and after gene silencing (gray columns) versus control cultures (represented by the dotted line). **c** Graphic illustrations comparing the proportion of cells at different cell cycle stages in cultures overexpressing *Uhrf2* (white columns) and after gene silencing (gray columns) versus control cultures (represented by the dotted line). ***p < 0.001; **p < 0.01; *p < 0.05 treated versus control

From our previous experiments, we concluded that *Uhrf* genes inhibit mesenchymal precursor differentiation. As mentioned above, skeletal progenitors that do not differentiate during limb development, undergo senescence and cell death. Therefore, we next explored whether *Uhrf* genes influenced the degenerative fate of the excess progenitors. For this purpose, changes in cell proliferation and death were analyzed in the micromass culture assay. After 2 days of culture, cell death was significantly regulated in gain-of-function and loss-of-function experiments (Fig. 7a). Over-expression of *Uhrf1* increased the number of dead cells more than three times, and its silencing decreased cell death by 25%. In turn, the overexpression of *Uhrf2* increased cell death by two-fold, and its knockdown decreased cell death by 35%. Changes in the intensity of cell death were accompanied by cell cycle arrest in the S-phase (Fig. 7b, c). Hence, the rate of cells in the S phase increased by 150% after *Uhrf1* overexpression and was reduced by 20% in loss-of-function experiments (Fig. 7b). The effect of *Uhrf2* on cell cycle progression was less consistent. *Uhrf2* overexpression increased the number of cells in S phase by 20% and silencing reduced S phase cells at a similar rate (Fig. 7c). Together, these findings are consistent with the arrest of cell cycle at S phase, as occurs in response to DNA damage.

### Transcriptional influence of *Uhrf* genes

UHRF factors act through the modulation of gene expression at the transcriptional level. Therefore, we next explored the influence of *Uhrf* genes in the transcription of a panel of genes associated with differentiation, proliferation, and apoptosis of limb mesenchymal progenitors (Table 1).

**Table 1:** Transcriptional analysis of limb progenitors subjected to gain-of-function and loss-of-function of either *Uhrf1* or *Uhrf2* genes

	cUhrf1	shUhrf1	cUhrf2	shUhrf2
*Differentiation markers*				
*Scx*	1.02 ± 0.11	**0.59** ± 0.06***	1.17 ± 0.20	**0.50** ± 0.09***
*Sox9*	**0.59** ± 0.07***	0.93 ± 0.09	**0.59** ± 0.08***	0.80 ± 0.15
*Senescence marker*				
*p21*	**6.18** ± 1.98*	**0.56** ± 0.07***	**2.36** ± 0.35**	**0.38** ± 0.08***
*Cell death markers*				
*Bcl2*	1.24 ± 0.10	1.12 ± 0.06	0.89 ± 0.07	1.01 ± 0.14
*Bak1*	**2.44** ± 0.51**	**0.59** ± 0.03***	**1.80** ± 0.34*	0.80 ± 0.10
*Bid*	1.45 ± 0.21	0.85 ± 0.06	1.24 ± 0.15	0.92 ± 0.15
*Bim*	0.99 ± 0.04	1.24 ± 0.12	0.91 ± 0.19	0.98 ± 0.12
*Bmf*	**1.72** ± 0.31*	0.94 ± 0.08	1.02 ± 0.13	0.89 ± 0.14

*Uhrf1*	**10.33** ± 2.88**	**0.44** ± 0.03***	**1.98** ± 0.35*	**0.69** ± 0.07***
*Uhrf2*	1.26 ± 0.12	0.95 ± 0.05	**10.31** ± 2.53**	**0.50** ± 0.04***

The panel of genes analyzed included markers for skeletogenic differentiation, senescence, and apoptosis

***p < 0.001; **p < 0.01; *p < 0.05 treated versus control

Statistic significant values are highlighted in bold

The dual differentiation of limb skeletal progenitors is regulated by a balance between *Sox9*, and *Scleraxis*, which are master chondrogenic and fibrogenic genes^31,32^, respectively. Both genes regulate the dichotomic differentiation of progenitors into cartilage or fibrous tissue. Increased chondrogenesis in knockdown experiments was preceded by downregulation of *Scleraxis*, while the upregulation of *Sox9* was not detectable until the overt differentiation of the micromass cultures (day 4 of culture). In contrast, the downregulation of *Sox9* in experiments of gain-of-function experiments was more precocious than the changes in the expression of *Scleraxis*.

The expression analysis of members of the *Bcl2* cell death gene family showed a significant regulation of *Bak1* in association with changes in the intensity of cell death. The overexpression of *Uhrf1* increased *Bak1* expression by 250%, while gene silencing decreased *Bak1* expression by half. *Uhrf2* functional experiments caused a similar but more moderate regulation of *Bak1*. Other members of the *Bcl2* gene family, were either not regulated or moderately regulated (Table 1).

To explore the influence of *Uhrf* genes in cell senescence we selected *p21* as the most conspicuous marker of embryonic developmental senescence. As shown in Table 1, *p21* was upregulated six-fold after *Uhrf1* overexpression and two-fold after *Uhrf2* overexpression. In turn, *p21* was significantly downregulated after the knockdown of either *Uhrf1* (0.5×) or *Uhrf2* (0.3×).

### Regulation of DNA methylation by Uhrf genes

**Fig. 8:**
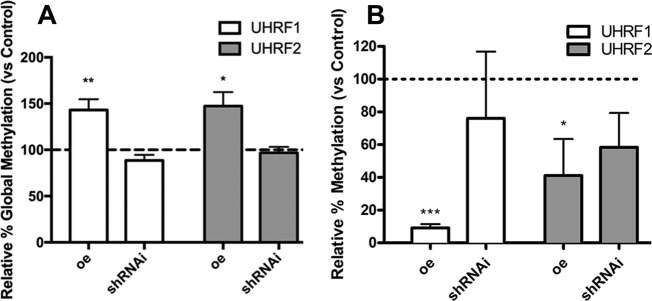
Changes in DNA methylation by UHRFs. **a** Chart showing differences in global methylation evaluated by ELISA between control progenitors and progenitors overexpressing (oe) or subjected to silencing (shRNAi) of *Uhrf1* (white columns) and *Uhrf2* (gray columns).The level of global methylation in control cultures was considered 100% and is represented by the dotted line. **b** Methylation level of CpG islands in the Bak1 promoter evaluated by MSRE-qPCR between control progenitors and progenitors overexpressing (oe) or subjected to silencing (shRNAi) of *Uhrf1* (white columns) and *Uhrf2* (gray columns). The level of CpG methylation in control cultures was considered 100% and is represented by the dotted line. ***p < 0.001; **p < 0.01; *p < 0.05 treated versus control

Considering the functional implication of UHRFs in genomic DNA methylation, we first analyzed changes in global methylation in micromass cultures subjected to *Uhrf* gene overexpression or silencing. Global methylation was significantly increased in 2-day cultures transfected with *Uhrf1* or *Uhrf2* genes (Fig. 8a), but was not modified at statistical significant levels when progenitors were subjected to *Uhrf*´s gene silencing. However, we detected a mild decrease in global methylation after *Uhrf1* gene silencing.

To address the functional significance of methylation in the regulation of cell death by *Uhrf* genes, we select *Bak1* to explore methylation changes in CpG islands of its promoter by MSRE-qPCR (Fig. 8b). Gain-of-function experiments of both *Uhrf1* or *Uhrf2* were followed by decreased methylation. The decrease was particularly intense after *Uhrf1* overexpression, and more moderated for *Uhrf2*. As observed for global methylation, gene silencing did not change at significant levels methylation of *Bak1* promoter.

### Functional redundancy of *Uhrf* genes

We first analyzed the influence of *Uhrf* genes on the expression of each other. As shown in Table 1, neither overexpression nor gene silencing of the *Uhrf1* gene regulated the expression of *Uhrf2* at significant levels. However, overexpression and silencing of *Uhrf2* were followed by a moderate regulation of *Uhrf1*.

We next analyzed by flow cytometry changes in cell death in combined transfections of overexpression plasmids and sh-RNA inhibitory constructs (Supplementary Fig. 2A, B). Transfection of *Uhrf2* in combination with shRNAi-*Uhrf1* neutralized the cell death promoting effect of Uhrf2 (Supplementary Fig. 2A). In these experiments, the intensity of cell death became similar to that observed after transfections with shRNAi-*Uhrf1* only. In a similar fashion, the inhibitory influence on cell death of sh-RNAi-*Uhrf2* was also abolished when it was co-transfected with *Uhrf1* (Supplementary Fig. 2B). Finally, the increased cell death induced separately by *Uhrf1* or *Uhrf2* lacked statistically significant differences with the level of cell death induced by double transfections with both *Uhrf* genes (Supplementary Fig. 2C). Together, these findings suggest common and complementary roles for both genes in the embryonic limb mesoderm.

## Discussion

Current knowledge of the role of UHRF proteins in developing vertebrates is scarce because mouse KO for *Uhrf1* die early in gestation^33^, and mice deficient in *Uhrf2* lack skeletal phenotype^18^. However, the implication of *Uhrf1* in the formation and growth of various organs has been observed in mutant zebrafish embryos^16^, and the influence of *Uhrf1* in cartilage maturation, has been demonstrated via a conditional knockout (*Uhrf1*-cKO) targeted to the limb mesoderm^17^. The skeletal phenotype of *Uhrf1*-cKO mice provided evidence for a role for UHRF1 in the elongation of appendicular bones during the postnatal period by regulating the growth and differentiation of the growth plates. Our study extends the function of these genes to the stages of digit skeletogenesis. Our study showed that *Uhrf* genes inhibit the chondrogenesis of skeletal progenitors. This function contrasts with the requirement of UHRF1 for the differentiation of postnatal chondrocytes grown in micromass culture^17^. These different effects emphasize the dependence of *Uhrf* genes on their functions of factors whose presence in the cells is related to the stage of differentiation. In the embryo, the expression domains of *Uhrf1* and *Uhrf2* specifically mark undifferentiated progenitors. Furthermore, in the case of *Uhrf1*, the level of expression in vitro becomes progressively reduced in parallel with the differentiation of the progenitors into chondrocytes. These observations support a role for *Uhrf* genes, especially *Uhrf1*, in the maintenance of progenitors in an undifferentiated state.

We identified *Sox9* and *Scleraxis*, as potential mediators for the antichondrogenic influence of UHRFs on the skeletal progenitors. *Sox9* belongs to the high-mobility group of chromatin regulators and plays a central role in the differentiation of skeletal progenitors^34–36^. Scleraxis, is a basic helix-loop-helix transcription factor structurally associated with Sox9 and is involved in the formation of fibrous connective tissues^31,35^. Sox9 promotes the onset of chondrogenic differentiation in the developing limb and its silencing leads to cell death of skeletal progenitors^34,37^. The function of SOX9 is modulated by epigenetic mechanisms via histone acetylation on chromatin^35^. Remarkably, in our study, the chondrogenic inhibition and increased cell death in *Uhrf* gene gain-of-function experiments involved a precocious downregulation of *Sox9*. In contrast, increased chondrogenesis in loss-of-function experiments were preceded by downregulation of *Scleraxis*. This finding suggests a double and complementary function of *Uhrf* genes via the activation or repression of the promoters of master genes that establish the fate of the skeletal progenitors.

UHRF proteins play pivotal functions in carcinogenesis modulating DNA methylation and the histone functional code of tumor cells^38–40^. Consistent with these facts, here we show that both *Uhrf* genes promote global methylation in skeletal progenitors. Changes induced in cancer cells by dysregulation of *Uhrf* genes include cell proliferation, cell senescence, apoptosis, increased metastatic potential, and increased sensitivity to DNA-damaging agents^14,15,40–44^. In most cases the function of UHRF proteins takes place in coordination with other chromatin-modifying proteins^44^. This feature explains that the function of UHRF proteins varies in a cell cycle-dependent and cell lineage-dependent manner.

The degenerative events accounting for interdigit remodeling occur in a sequential fashion. In the 12–24 h preceding the onset of massive apoptosis, the interdigital cells undergo proliferation arrest^24^, and intense DNA damage^5^. These changes are next followed by senescence^4^ and massive apoptosis to accomplish interdigit removal. Between id 5.5 and id 6, we observed a partial decrease in the interdigital expression of *Uhrf1* that correlated with the downregulation of FGF genes at the end of limb morphogenesis. In subsequent stages of degeneration (from id 6.5 to id 8) both UHRF1 and UHRF2 maintained elevated protein and transcriptional expression levels, suggesting an active participation of both UHRF proteins in tissue regression.

Our functional approaches established the implication of both *Uhrf* genes in the regulation of the cell death of skeletal progenitors, and in the expression of *p21*, a gene that plays a pivotal role in embryonic developmental senescence^4^. Additionally, the overexpression of *Uhrf* genes arrested the cell cycle in S phase, a characteristic feature of senescent cells associated with DNA damage. Cell death appeared significantly upregulated in progenitors overexpressing either *Uhrf1* or *Uhrf2*, and significantly reduced in loss-of-function experiments of either gene. UHRFs have been implicated in the regulation of apoptosis in tumoral systems by distinct mechanisms, including the transcriptional regulation of tumor suppressor genes^38^, selective regulation of pro-apoptotic genes^14,45^, the induction of global hypomethylation leading to changes in tumor radiosensitivity^39^, or by reducing the capacity to repair DNA damage^46^. Remarkably, in our experimental system the intensification and attenuation of cell death correlated with changes in the expression of *Bak1*, a characteristic proapoptotic member of the *Bcl2* gene family with a demonstrated role in the regression of the interdigits^47^. Furthermore, up-regulation of *Bak1* was associated with hypomethylation of the CpG islands of its promoter.

In normal cells, UHRF1 participates in the regulation of the cell cycle, and in cooperation with the DNA methyl transferase 1 (DNMT1), UHRF1 maintains the DNA methylation status of dividing cells. It has been proposed that *Uhrf1* plays a primary role in cell senescence regulating the expression DNTM1^48^. In our system, DNMT1 was not regulated by *Uhrf1* or *Uhrf2* (unpublished observation), but, in turn, we detected an intense regulation of the cell senescence master gene *p21* indicative of the direct implication of these genes in cell senescence. A similar regulation of *p21* by *Uhrf1* has been reported in a number of systems^44,49^. However, in some tumoral cell lineages, UHRF1 and UHRF2 exerted negative transcriptional influence on the expression of *p21*^50^. This discrepancy is explained by the importance of specific cofactors that, together with UHRFs, form heteromeric binding complexes in the promotors of target genes, modulating their functions in a cell-cycle and cell-lineage-dependent manner^44^.

In conclusion, our study uncovers a new level of regulation of interdigital apoptosis and cell senescence upstream of the components of the intrinsic pathway responsible for executing cell death in embryonic systems. Furthermore, our findings indicate that the balance between cell differentiation and cell stemness may be a central step in the initiation of the so-called “programmed cell death” associated with embryonic morphogenesis. The epigenetic functional profile of *Uhrf* genes in most studied systems, together with the changes in DNA methylation observed in our functional experiments, suggests that the structural organization of the chromatin may be a critical factor in the regulation of embryonic cell death and cell senescence.

## Supplementary information

Supplementary figure1

Supplementary figure 2

Supplementary figure 3

supplementary figure legends
