# Supplementary figures and images for: UHRF genes regulate programmed interdigital tissue regression and chondrogenesis in the embryonic limb
Source: Cell Death Dis. 2019 Apr 25;10(5):347. doi: 10.1038/s41419-019-1575-4 (PMC6484032; doi:10.1038/s41419-019-1575-4)

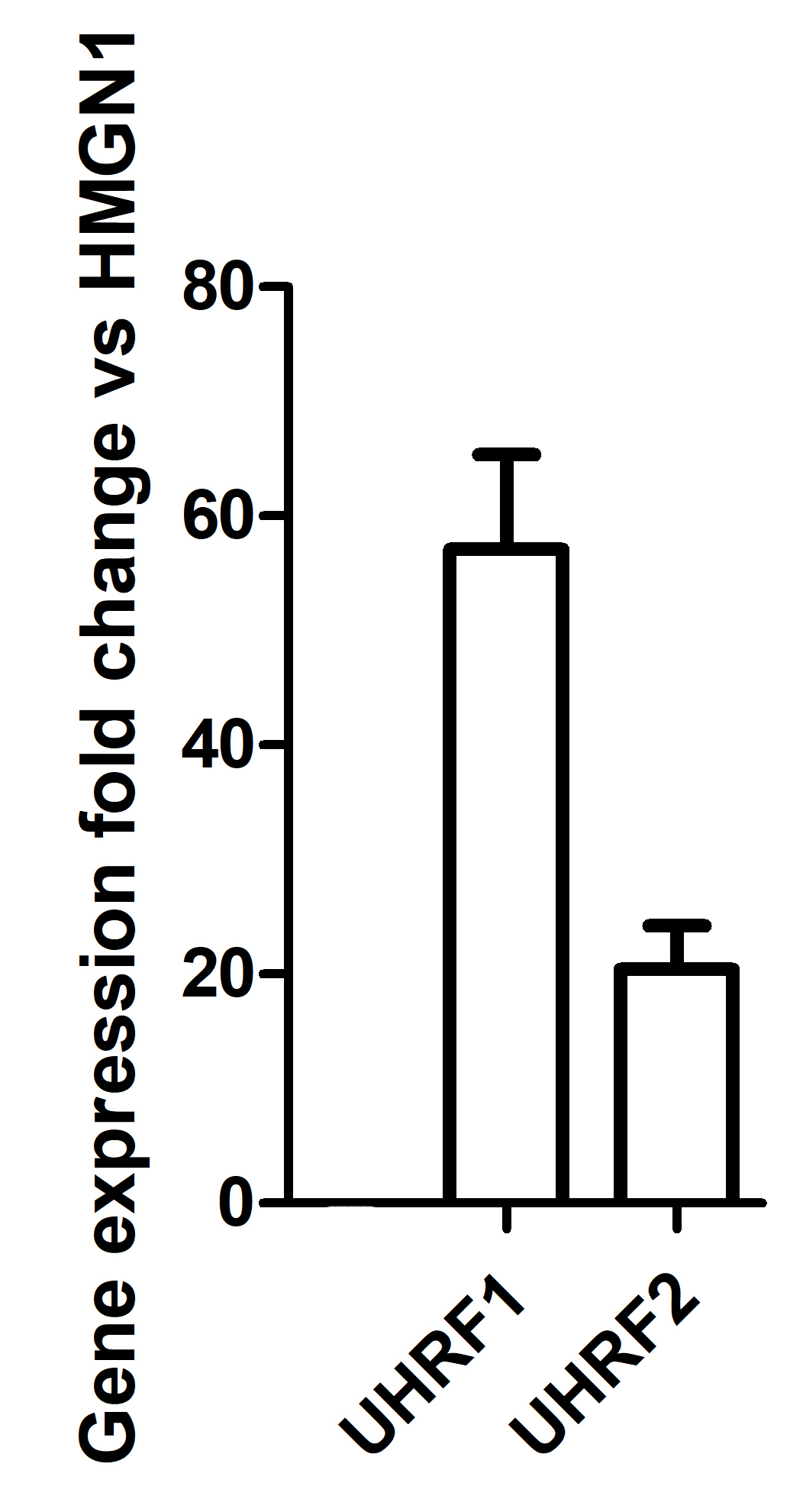

Supplement: Supplementary file 1 — Supplementary figure1 [file 41419_2019_1575_MOESM1_ESM.tif]

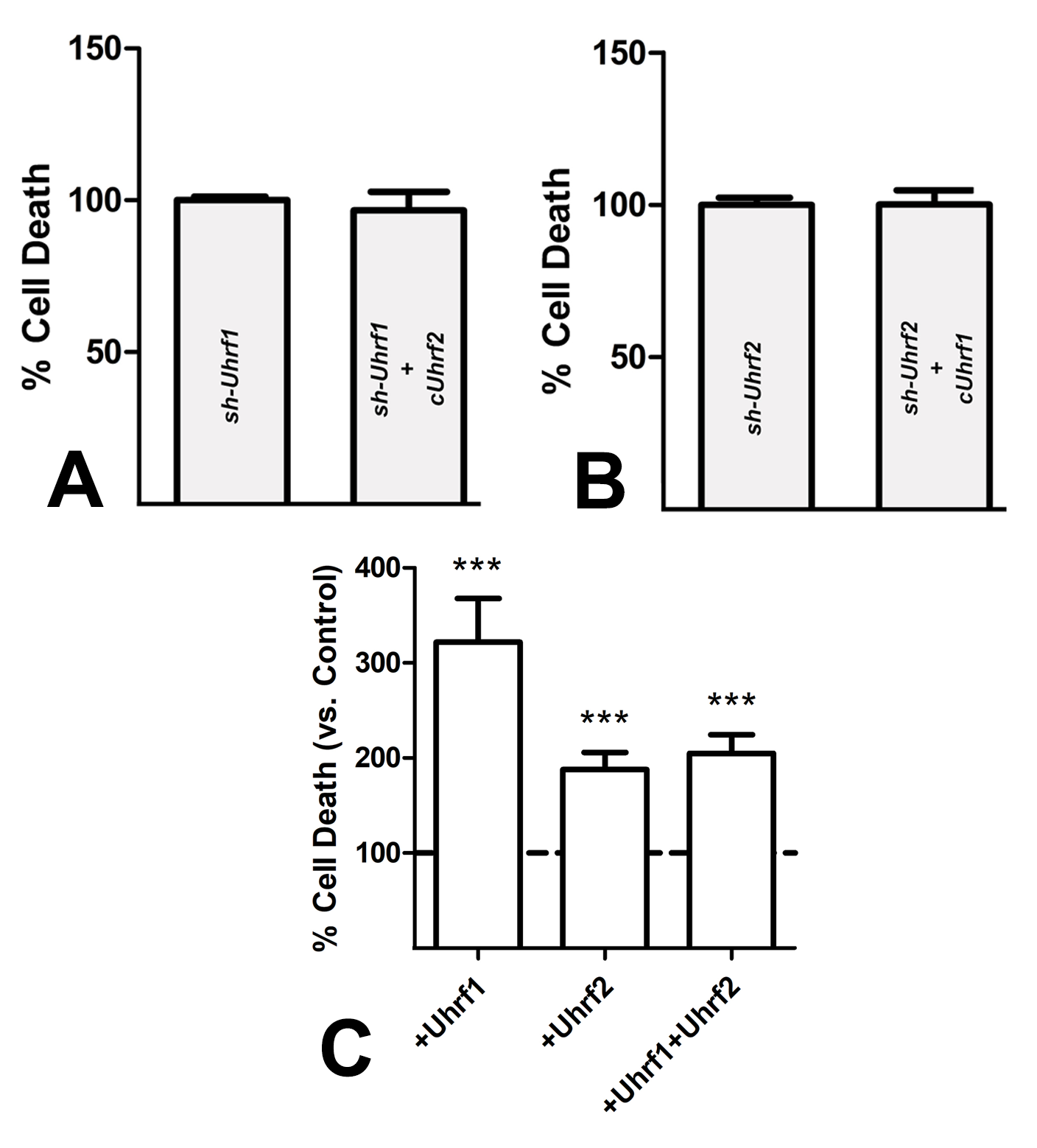

Supplement: Supplementary file 2 — Supplementary figure 2 [file 41419_2019_1575_MOESM2_ESM.tif]

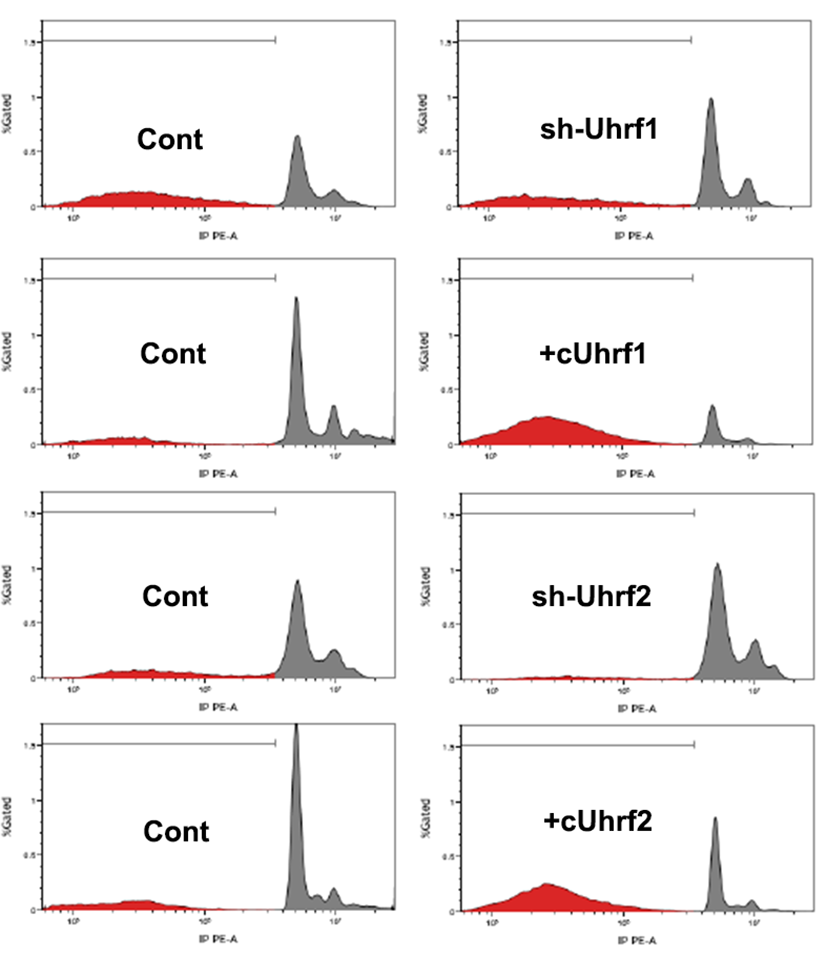

Supplement: Supplementary file 3 — Supplementary figure 3 [file 41419_2019_1575_MOESM3_ESM.tif]
