# Supplementary material for: UHRF genes regulate programmed interdigital tissue regression and chondrogenesis in the embryonic limb
Source: Cell Death Dis. 2019 Apr 25;10(5):347. doi: 10.1038/s41419-019-1575-4 (PMC6484032; doi:10.1038/s41419-019-1575-4)
Supplement: Supplementary file 4 — supplementary figure legends [file 41419_2019_1575_MOESM4_ESM.docx]

**Supplementary Fig. 1**.- Comparative q-PCR analysis showing differences in the expression levels of *Uhrf1* and *Uhrf2* versus HMGN1 in interdigital tissue samples at id 6.

**Supplementary Fig. 2**.- A, chart comparing the rate of cell death of progenitors subjected to *Uhrf1* silencing only (first column) in relation to control cultures transfected with empty vectors, in comparison with progenitors, double transfected with sh-RNAi-Uhrf1 and *Uhrf2* (second column). For comparison purposes, the rate of cell death of progenitors transfected with sh-RNAi-Uhrf1 was considered 100%. B, chart showing the results of a similar analysis comparing the intensity of cell death in progenitors subjected to *Uhrf2* silencing only (first column, value 100%) or, with double transfection with sh-RNAi-Uhrf2 and *Uhrf1* (second column).

**Supplementary Fig. 3**.- Iodide propidium flow cytometry plots to illustrate differences in the intensity of cell death in representative experiments of gain- and loss-of-function.
